# Uptake and associated factors of six multi-month scripting/appointment spacing differentiated service delivery model of care among stable clients on antiretroviral therapy in Southern Ethiopia

**DOI:** 10.1371/journal.pone.0310400

**Published:** 2024-09-12

**Authors:** Fasika Merid, Tamirat Gezahegn Guyo, Simegn Wagaye, Mesele Mekuria, Abraham Anbesie, Temesgen Mohammed Toma

**Affiliations:** 1 Department of Public Health, Arba Minch College of Health Sciences, Arba Minch, Ethiopia; 2 Department of Public Health Emergency Management, South Ethiopia Region Public Health Institute, Jinka, Ethiopia; FHI 360, ZAMBIA

## Abstract

**Background:**

Differentiated service delivery models have been developed to accommodate the rising number of stable antiretroviral therapy clients and to maintain improvements in health outcomes and care retention. Ethiopia adopted the appointment spacing model and has had notable successes in implementing it. However, with the implementation of the six multi-month scripting/appointment spacing model in Ethiopia, little is known about the uptake and its associated factors. Therefore, this study aimed to assess the uptake and associated factors of the six multi-month scripting/appointment spacing differentiated service delivery model of care among stable clients on antiretroviral therapy in Southern Ethiopia.

**Methods:**

A hospital-based cross-sectional study was conducted among 419 stable clients on antiretroviral therapy in southern Ethiopia from June 22 to September 29, 2023. A systematic sampling technique was used to select the study participants. Using a structured questionnaire, socio-demographic, health service delivery, behavioral, and clinical-related data were collected. The collected data were entered into Epi Data version 3.1 and analyzed using Stata version 14. Variables with a P-value <0.05 in the multivariable logistic analysis were considered statistically significant. Multicollinearity and model fitness were checked using the variance inflation factor and the Hosmer and Lemeshow goodness of fit tests, respectively.

**Results:**

The uptake of the six multi-month scripting/appointment spacing differentiated service delivery model of care was 63.25% (95% confidence interval (CI): 58.61%, 67.88%). Missed appointment (Adjusted Odds Ratio (AOR): 1.91 (95% CI: 1.13, 3.25)), distance to antiretroviral therapy facility (AOR: 2.90 (95% CI: 1.67, 5.04)), duration on antiretroviral therapy (AOR: 2.21 (95% CI: 1.34, 3.64)), and intermediate social support (AOR: 2.02 (95% CI: 1.29, 3.17)) and strong social support (AOR: 2.71 (95% CI: 1.23, 5.97)) were factors significantly associated with the uptake.

**Conclusion:**

The uptake of six multi-month scripting/appointment spacing differentiated service delivery models of care was six out of ten clients on antiretroviral therapy. To further improve the uptake, a precise intervention on the identified associated factor is required.

## Introduction

Globally in 2023, 39.9 million people were living with the Human Immunodeficiency Virus (HIV), 1.3 million became newly infected, 630, 000 died from Acquired Immune Deficiency Syndrome (AIDS)-related illnesses, and 30.7 million people were accessing antiretroviral therapy (ART) [1]. Sub-Saharan Africa is the most affected part of the world; 26 million people are living with HIV, representing two-thirds of the global burden [2]. According to the Ethiopian Public Health Institute (EPHI) 2022 HIV-related estimates and projections, 609,349 people were living with HIV, 10,567 acquired HIV, and 10,421 people died related to AIDS in Ethiopia. The national HIV prevalence among adults living with HIV was 0.9% in 2022 [3].

The Joint United Nations Programme on HIV/AIDS launched the 95-95-95 targets, which aim to diagnose 95% of all HIV-positive individuals, provide ART for 95% of those diagnosed, and achieve viral suppression for 95% of those treated by 2030 [4]. In Ethiopia, it is estimated that 84% of people living with HIV (PLHIV) know their HIV status; 83% are confirmed to be receiving ART, and among adults receiving ART, 96% were virally suppressed [5].

The delivery of HIV care has been based on a “one-size-fits-all” clinic-based model, largely undifferentiated for individual needs. As recommendations evolve towards initiating ART for all PLHIV, regardless of clinical and immunological status, the HIV programs are challenged by a growing cohort of patients who have been on treatment for several years and the need to expand timely access to ART for those who have yet to start [6]. An approach that differentiates clients with HIV according to broader definitions of care, and treatment was more likely to succeed [7].

Differentiated Service Delivery (DSD) is “a client-centered approach that simplifies and adapts HIV services across the cascade in ways that both serve the needs of people living with HIV better and reduce unnecessary burdens on the health system” [8]. Nations need to adopt and adapt the existing DSD models to address both the increasingly diverse needs of PLHIV and the health system’s capacity and constraints [7]. The Appointment Spacing Model (ASM) is an approach that adapts and simplifies HIV care services to reduce frequent clinic visits and medication pick-ups, free up healthcare resources, and improve the efficiency of care and management for patients [9]. In sub-Saharan Africa, Ethiopia is the first country to implement six multi-month scripting (MMS)/ASM at scale and adopted it for HIV care in 2017 considering the sociocultural situation, degree of awareness, stigma and discrimination, resource demand, and its sustainability [10–13]. In the ASM stable, clients are appointed every six months for clinical visits and medication refills [10, 12].

The magnitude of uptake of ASM in different African countries other than Ethiopia ranges from 10.3% to 82.1% [14–18], whereas it is 50.1% in Ethiopia [19]. Appointment spacing model (ASM) implementation of ART delivery improves service quality, reduces health care costs, improves health outcomes, and accelerates the achievement of the 95-95-95 target through offloading workload from overburdened health facilities, and improved adherence and retention [10, 12]. Studies also showed the wide range of benefits of having less frequent visits to health facilities for clients as well as providers, which includes decreased direct costs in transportation; decreased indirect costs in lost wages; less time spent traveling to the health facility, particularly if they lived far away; and less time waiting and reduced workload for providers [20–23]. Thus, ASM was perceived as an ideal dispensing interval for ART with clinic decongestion, reduced provider workload, increased ability of providers to focus on unstable clients, and reduced burden of cost related to accessing the clinic frequently [23].

Evidence from previous studies showed that residence, monthly income, catchment area of an ART facility, alcohol use, social support, viral load, missed clinical appointment, WHO clinical staging, duration on ART, and baseline regimen change were factors associated with the uptake of ASM [19, 24–27].

Ethiopia has had notable successes in implementing ASM. Successful implementation and scale-up have also been associated with high viral suppression rates among clients, despite fewer clinical visits. Further strengthening and maximizing the implementation of ASM and optimizing the collaboration between facility and community services is necessary to continue progress toward epidemic control [5]. However, with the implementation of six month MMS/ASM in Ethiopia, little is known about the uptake and its associated factors, and no study was conducted in the study settings. Moreover, the previous study measured the uptake of ASM without taking into account the duration of ART for at least a year [19]. Thus, this study aimed to assess the uptake and associated factors of the six multi-month scripting/appointment spacing differentiated service delivery model of care among stable clients on antiretroviral therapy. The findings of this study will be useful to improve the quality of services and promote the sustainability of HIV treatment programs, particularly in resource-constrained countries like Ethiopia. In addition, the study may help program planners set intervention strategies for the factors associated with the uptake of ASM based on the evidence. Moreover, it will contribute to achieving the three 95s global targets.

## Method and materials

### Study setting and period

The study was conducted at Arba Minch General Hospital, Jinka General Hospital, and Wolaita Sodo University Comprehensive Specialized Hospital, Southern Ethiopia, from June 22 to September 29, 2023. Arba Minch town is the administrative center of the Gamo zone and the South Ethiopia regional state. Jinka town is the administrative center of Ari Zone and the South Ethiopia regional state. Wolaita Sodo town is the administrative center of Wolaita zone and South Ethiopia regional state. The hospitals are providing different services, like outpatient department services, inpatient department services, emergency services, maternal and child health services, dental treatment, ophthalmic services, and follow-up services, to the community in their catchment area and nearby Woredas and zones. In these three public hospitals, during the study period, there were 4,891 HIV-positive adults on active ART follow-up, of whom 3,905 were stable clients.

### Study design

A hospital-based cross-sectional study was conducted.

### Study population

Following the appropriate classification of clients based on the category, patients were informed and gave verbal consent to the service delivery frequency. Eligibility for categories and adherence were monitored continuously according to the ASM of the HIV care classification tool for clients [10].

All stable adult clients with HIV on ART in Arba Minch General Hospital, Jinka General Hospital, and Wolaita Sodo University Comprehensive Specialized Hospital, Southern Ethiopia, were the source population. All systematically selected stable adult clients with HIV on ART and visiting ART clinics in selected public hospitals in southern Ethiopia during the data collection period, and those who fulfilled inclusion criteria were the study population. Stable adult clients aged ≥ 18 years with HIV on ART were the inclusion criteria, and clients who were referred from other ART health facilities with incomplete information and those with incomplete ART intake forms and clinical data were excluded from the study.

### Sample size determination and sampling technique

The sample was calculated by using the formula to estimate a single population proportion with the assumptions of 95% confidence interval Zα/2 = 1.96, proportion (P) = 50.1% from the previous study [19], margin of error (d) = 0.05, and 10% non-response rate.

n = (Zα/2)^2^ p (1-p) / d^2^ = (1.96)^2^ 0.501(0.499) / 0.05^2^ = 384

By adding 10%, the final required sample size was 422.

Currently, public health facilities are implementing ASM for HIV care. A high-load facility, Arba Minch General Hospital, Jinka General Hospital, and Wolaita Sodo University Comprehensive Specialized Hospital, were selected purposively. The total sample size was allocated proportionally to the selected public hospitals. Study participants were selected using a systematic sampling technique of every three K interval from each hospital.

### Data collection tool, personnel, and procedure

A structured questionnaire was developed in English after reviewing all relevant literature and ART intake and follow-up forms [19, 24–27] and then translated into Amharic. The data collection tool consists of socio-demographic characteristics, health service delivery-related characteristics, knowledge and behavioral-related characteristics, and clinical-related characteristics. The data was collected by six BSc nurses and supervised by three public health professionals. Data were collected through interviews and observation of clients’ medical charts.

### Study variable

#### Dependent variable

Uptake of ASM (Yes, coded “1” and No, coded “0”).

#### Independent variable

Socio-demographic and economic characteristics (age, sex, residence, marital status, educational status, occupation, religion, ethnicity, and monthly income), Health service delivery characteristics (type of health facilities, ART facility catchment, and distance to ART facility), Knowledge and behavioral characteristics (knowledge about ART, khat chewing, alcohol use, frequency of condom use, and number of the sexual partner), and Clinical characteristics (duration on ART, WHO clinical stage, viral load, CD4 count, regimen change, INH prophylaxis status, CPT status, missed clinical appointment, disclosure status, and social support).

#### Operational definitions

Stable is defined as being on ART for at least one year; no adverse drug reactions requiring regular monitoring; a good understanding of lifelong adherence; evidence of treatment success (i.e., two consecutive VL measurements < 1000 copies/mL or rising CD4 cell counts, or CD4 counts above 200 cells/mm3); no acute illness; not pregnant or breastfeeding [10, 11].

Good knowledge of ART: study participants who scored median and above value from the questions to assess knowledge related to ART.

Poor knowledge of ART: study participants who scored below the median value from the questions to assess knowledge related to ART.

Alcohol use: Item responses on the Cut down, Annoyed, Guilty, and Eye-opener (CAGE) questions are scored 0 for "no" and 1 for "yes" answers, with a higher score being an indication of alcohol problems. A total score of two or greater is considered clinically significant [28].

Social support: The Oslo Social Support Scale (OSSS-3) consists of a three-item scale that has a sum score that ranges from 3 to 14 (poor social support = 3–8, intermediate social support = 9–11, strong social support = 12–14) [29].

#### Data quality assurance

A pretest using 5% of the sample size was conducted at Sawla General Hospital before actual data collection, and adjustments were made to the data collection tool. A two-day training was given to data collectors and supervisors before actual data collection. Daily-filled questionnaires were checked by supervisors for completeness and consistency, and corrections were made accordingly during the entire data collection period.

#### Data processing and analysis

The collected data were checked for completeness and consistency, then entered, coded, edited, and cleaned using Epi-Data version 3.1 and exported to Stata version 14 for further analysis. Descriptive statistical analysis, including frequencies with percentage, and median with interquartile range (IQR) was performed. A binary logistic regression model was fitted to identify factors associated with the outcome variables. Variables in bivariable logistic analysis with a p-value <0.25 were used as candidates for multivariable logistic regression analysis. The model was built using the backward stepwise elimination method. Variables with a P-value <0.05 in the multivariable logistic analysis were considered statistically significant. Multicolinearity was checked using the variance inflation factor (mean VIF = 1.04). The model fitness was checked using the Hosmer and Lemeshow goodness of fit test (Prob > chi2 = 0.8245). An adjusted odds ratio (AOR) with a 95% confidence interval was used to show the presence and strength of the association and to identify statistically significant associated factors.

#### Ethical consideration

An ethical clearance letter was obtained from the institutional review board of Arba Minch College of Health Science with reference number AMCHS/01/2023/01. The official support letter was obtained from the Arba Minch College of Health Science research and community service directorate office. Confidentiality was maintained using a code. Informed written consent was obtained from study participants before data collection.

## Results

### Socio-demographic and economic characteristics

The study response rate was 99.3%. The median age of the study participants was 40 years, with an IQR of 27–53 and 170 (40.6%) of the adults’ age was between 35 and 44 years. More than half (57%) of the respondents were female and more than three-fourths (76.1%) of the stable adults who participated in the study were urban residents. Respondents who attended primary education account for nearly one-third, 158 (37.7%) of the total respondents, and those who attended college and above were 55 (13.1%). More than two-thirds, 288 (68.7%) of the respondents had a monthly income of 2500 birr and below, and only 39 (9.3%) had an income of 5001 and above (Table 1).

**Table 1:** Socio-demographic and economic characteristics of stable clients with HIV on ART in Southern Ethiopia (n = 419).

Variables	Categories	Frequency	Percent
Age (in years)	18–24	15	3.6
	25–34	98	23.4
	35–44	170	40.6
	≥45	136	32.4
	Median (IQR) 40 (27–53)		
Sex	Male	180	43.0
	Female	239	57.0
Residence	Urban	319	76.1
	Rural	100	23.9
Marital status	Single	31	7.4
	Married	246	58.7
	Divorced/Separated	72	17.2
	Widowed	70	16.7
Educational status	No formal education	102	24.4
	Primary education	158	37.7
	Secondary education	104	24.8
	College and above	55	13.1
Occupational status	Government employee	90	21.5
	House wife	97	23.2
	Merchant	88	21.0
	Daily laborer	72	17.2
	Student/Farmer	40	9.5
	Other*	32	7.6
Religion	Orthodox	231	55.1
	Protestant	153	36.5
	Muslim	25	6.0
	Other**	10	2.4
Ethnicity	Gamo	70	16.7
	Goofa	63	15.0
	Wolaita	155	37.0
	Ari	40	9.6
	Amhara	53	12.7
	Other***	38	9.0
Monthly income (Ethiopian birr)	≤2500	288	68.7
	2501–5000	92	22.0
	≥5001	39	9.3

*Driver, NGO, Self-employed, Evangelist, Pastoralist, Unemployed, Retired

**Catholic, Apostolic

***Konso, Bena, Zeyise, Sidama, Derashe, Tigrai, Kembata, Gurage, Oromo, Basketo, Malee, Hamer, Mursi

### Health service delivery, knowledge about ART, and behavioral characteristics

Out of 419 study participants, 320 (76.4%) travel ≤ 60 minutes to reach ART clinics in order to receive care. Regarding knowledge about ART, forty-six percent (193) of the respondents had poor knowledge. Nearly twenty percent, 68 (16.2%), had alcohol use problems. More than half, 219 (52.3%) of the study participants have never utilized condom, and 257 (61.3%) of the total respondents had a sexual partner in the last six months before the study (Table 2).

**Table 2:** Health service delivery, knowledge about ART, and behavioral characteristics of stable clients with HIV on ART in Southern Ethiopia (n = 419).

Variables	Categories	Frequency	Percent
Type of health facilities	General Hospital	280	66.8
	Comprehensive Specialized Hospital	139	33.2
ART facility catchment	Within the catchment	326	77.8
	Out of the catchment	93	22.2
Distance to ART facility (in minutes)	≤60	320	76.4
	>60	99	23.6
Knowledge on ART	Poor	193	46.1
	Good	226	53.9
Ever khat chew	Yes	76	18.1
	No	343	81.9
Alcohol use problem	Yes	68	16.2
	No	351	83.8
Condom use	Always	63	15.0
	Sometimes	137	32.7
	Never	219	52.3
Sexual partner in the last 6 months	Yes	257	61.3
	No	162	38.7
Number of sexual partners in the last 6 months (n = 257)	One sexual partner	234	91.1
	≥2 sexual partner	23	8.9
Who is a sexual partner (n = 257)	Husband/wife	232	90.3
	Commercial sex worker	17	6.6
	Bar ladies	9	3.5
	Others*	8	3.1

*Girlfriend, Boyfriend

#### Clinical characteristics

Out of 419 study participants, 329 (78.5%) were on ART for >5 years. The recent viral load among study participants was undetectable (≤50 copies/ml) in 94.8%. Currently, all (100%) of study participants are on the TDF+3TC+DTG ART regimen, and more than three-fourths (88.3%) of study participants baseline regimen was changed. Nearly one-fifth (18.9%) of study participants ever missed their clinical appointment. Three hundred seventy-nine (90.4%) disclosed their HIV status, and one out of ten (9.8%) study participants had strong social support (Table 3).

**Table 3:** Clinical characteristics of stable clients with HIV on ART in Southern Ethiopia (n = 419).

Variables	Categories	Frequency	Percent
Duration on ART (in years)	≤5	90	21.5
	>5	329	78.5
Baseline WHO clinical stage	I	212	50.6
	II	82	19.6
	III	108	25.8
	IV	17	4.0
Recent viral load	Undetectable	397	94.8
	Detectable	22	5.2
Baseline CD4 count	<500 cell/mm^3^	347	82.8
	≥500 cell/mm^3^	72	17.2
Baseline ART regimen	d4T-3TC-NVP	22	5.3
	d4T-3TC-EFV	10	2.4
	AZT-3TC-NVP	94	22.4
	AZT-3TC-EFV	27	6.4
	TDF-3TC-EFV	193	46.1
	TDF+3TC+NVP	24	5.7
	TDF+3TC+DTG	49	11.7
Regimen Change	Yes	370	88.3
	No	49	11.7
INH prophylaxis	Completed	395	94.3
	Not complete/Not given	24	5.7
CPT prophylaxis	Ongoing taking	31	7.4
	Not complete	15	3.6
	Completed	261	62.3
	Not given at all	112	26.7
Missed clinical appointment	Yes	79	18.9
	No	340	81.1
Disclosure status	Yes	379	90.4
	No	40	9.6
Social support	Poor	215	51.3
	Intermediate	163	38.9
	Strong	41	9.8

#### Uptake of six month MMS/ASM

**Fig 1:**
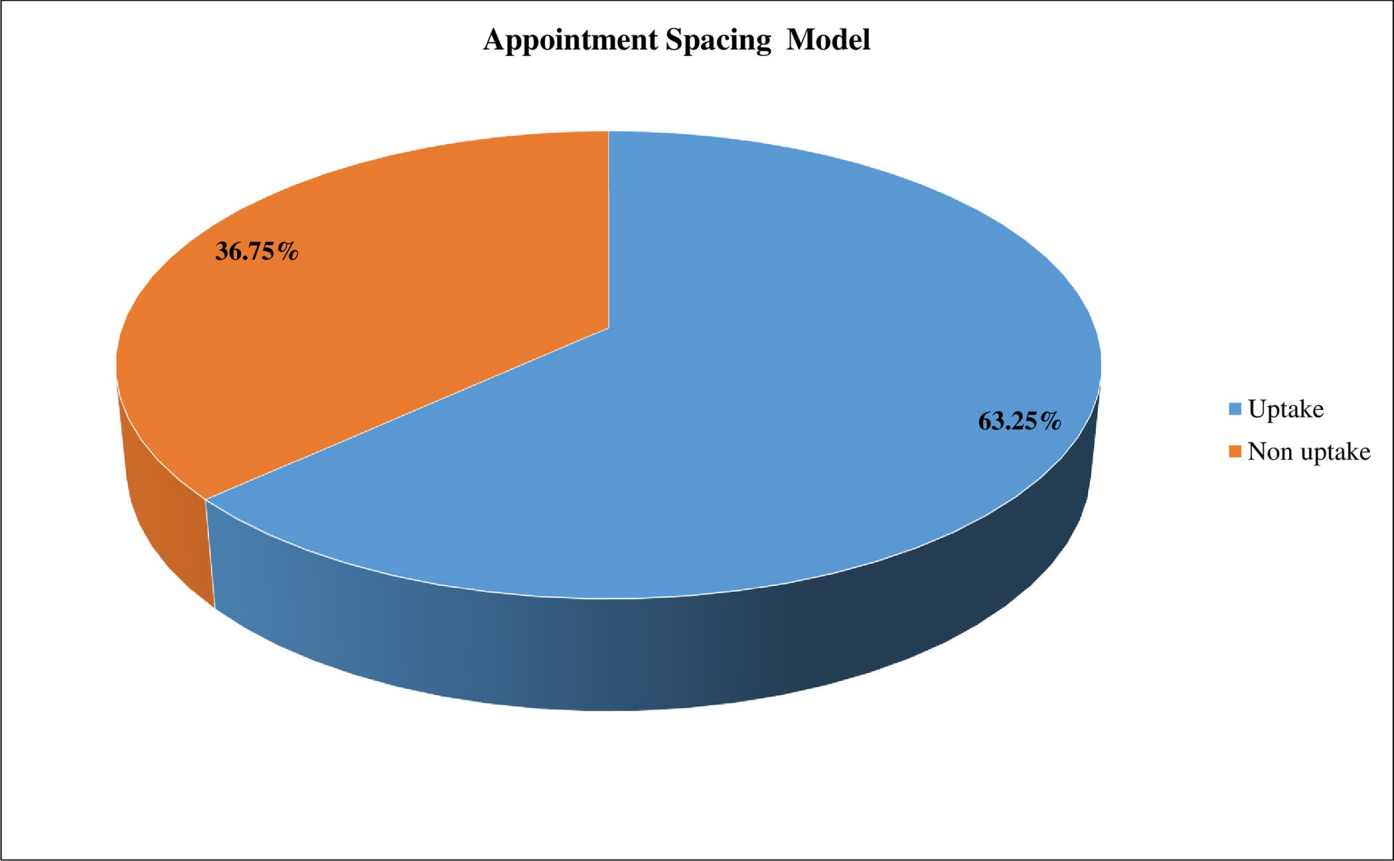
Uptake of ASM of care status of stable clients on ART in Southern Ethiopia (n = 419).

**Fig 2:**
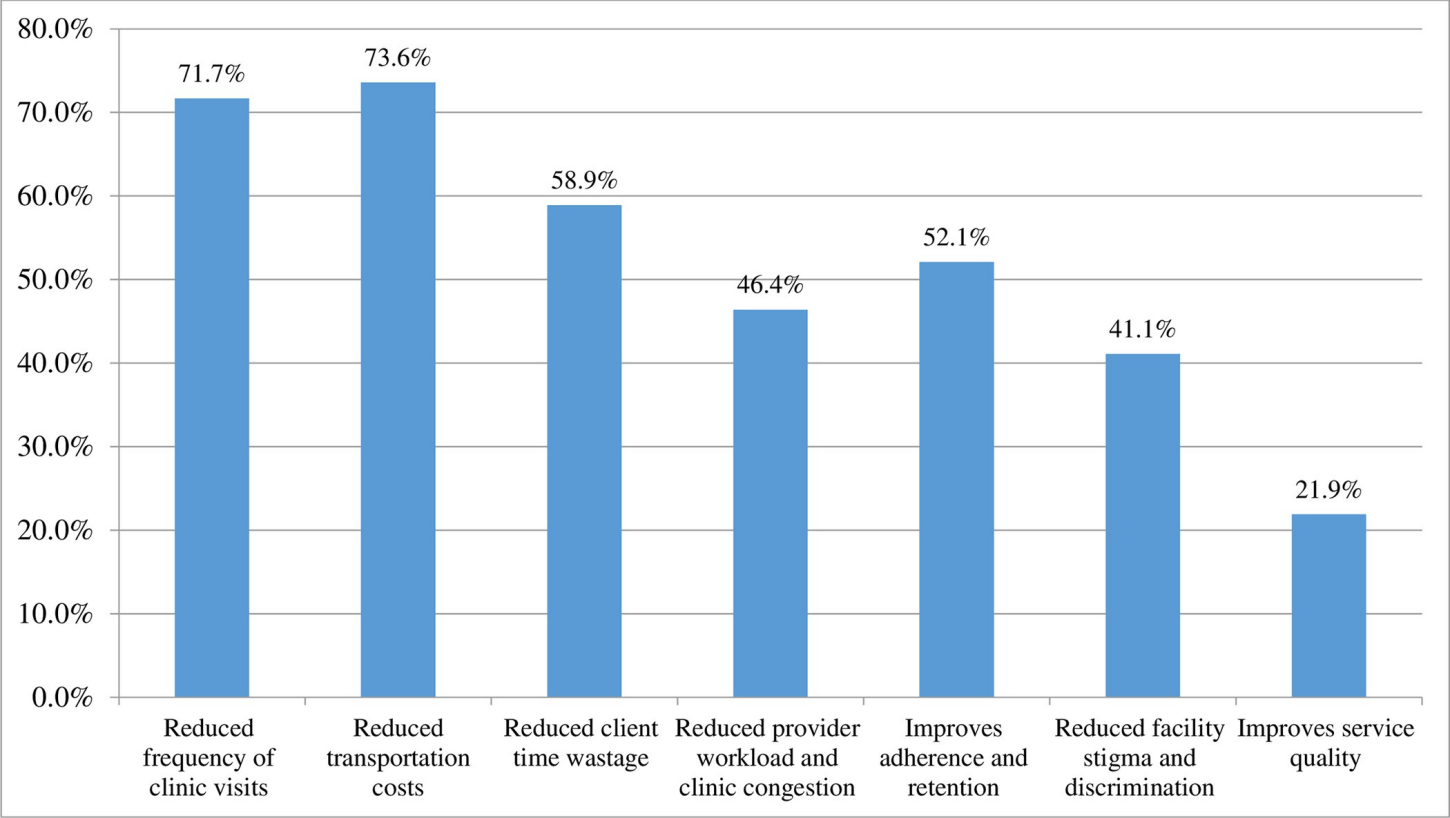
Reason to uptake ASM of care among stable clients on ART in Southern Ethiopia (n = 419).

**Fig 3:**
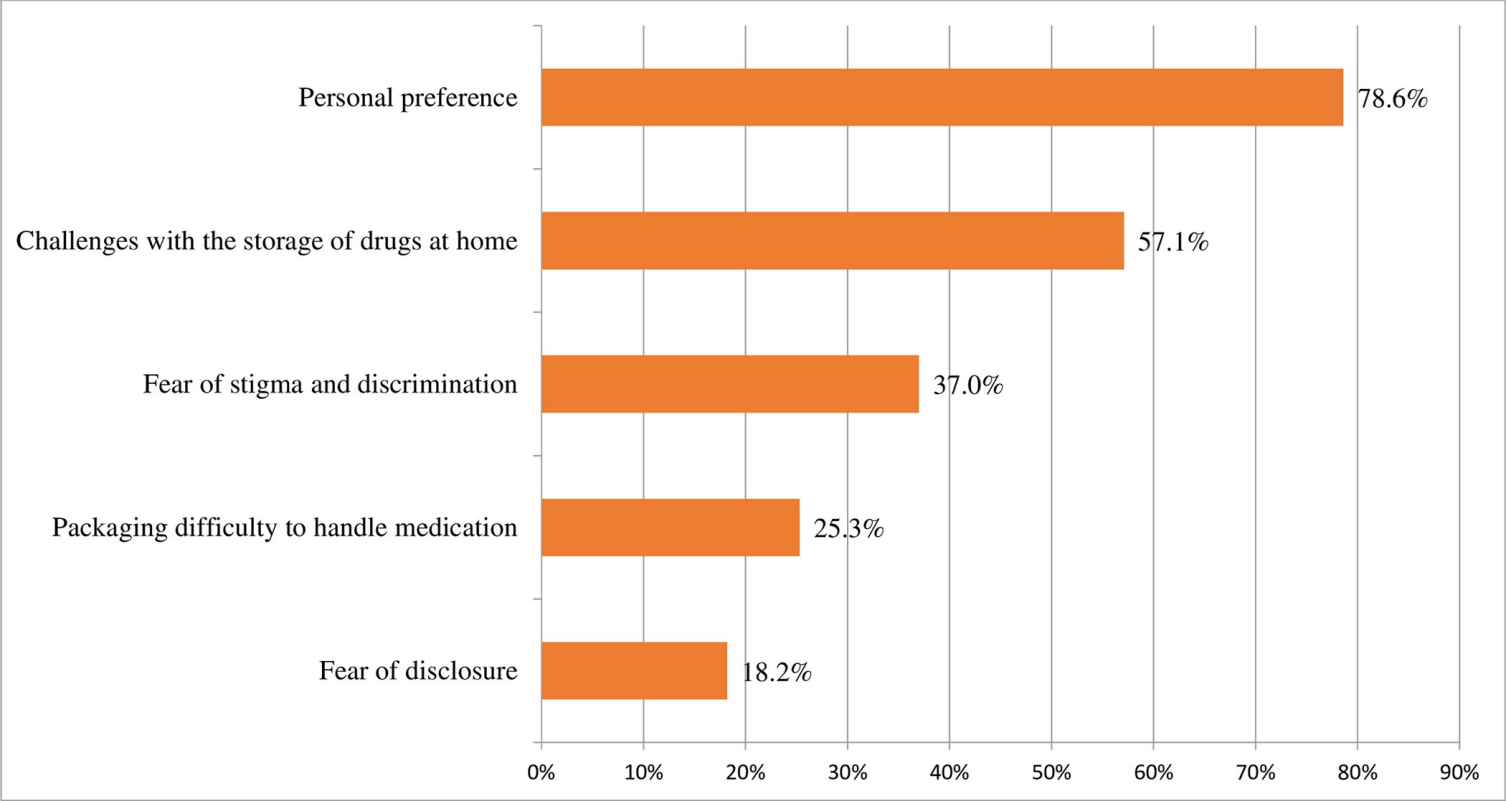
Reason to non-uptake ASM of care among stable clients on ART in Southern Ethiopia (n = 419).

The study found that the uptake of six-month MMS/ASM was 63.25% [95% CI (58.61%, 67.88%)] among stable clients on ART (Fig 1). The most common reason to uptake six-month MMS/ASM was reduced transportation costs (73.6%), reduced frequency of clinic visits (71.7%), reduced client time wastage (58.9%), and improves adherence and retention (52.1%) (Fig 2). The major reasons for non-uptake of six-month MMS/ASM were personal preference (78.6%) and challenges with the storage of drugs at home (57.1%) (Fig 3).

### Factors associated with uptake of six month MMS/ASM

In bivariable logistic regression analysis, age, residence, marital status, educational status, occupation, monthly income, ART facility catchment, distance to ART facility, duration on ART, WHO clinical stage, recent viral load, regimen change, INH prophylaxis, CPT prophylaxis, missed appointment, disclosure status, and social support were identified as having a statistically significant association with six-month MMS/ASM uptake with a P-value of <0.25. In multivariable analysis, distance to ART facility, duration on ART, missed clinical appointment, and social support were significantly associated with six-month MMS/ASM uptake with a p-value <0.05. Stable adults on ART who were living at a distance greater than 60 minutes from the ART service facility were nearly three times (AOR = 2.90; 95% CI: 1.67, 5.04) more likely to uptake the six month MMS/ASM of care compared to their counterparts. The odds of uptake of the six month MMS/ASM of care were 2.21 times (AOR = 2.21; 95% CI: 1.34, 3.64) higher among stable HIV-positive adults who stayed more than five years on ART than those who stayed below five years. Adults who never missed clinical care appointments had doubled (AOR = 1.91; 95% CI: 1.13, 3.25) odds of the six month MMS/ASM of care uptake when compared to those who ever missed an appointment. Getting intermediate and strong social support increased the likelihood of six month MMS/ASM of care uptake by two times (AOR = 2.02; 95% CI: 1.29, 3.17) and nearly three times (AOR = 2.71; 95% CI: 1.23, 5.97) among stable adults, respectively, than adults with poor social support (Table 4).

**Table 4:** Multivariable logistic regression analysis for factors associated with uptake of ASM of care among stable clients on ART in Southern Ethiopia (n = 419).

Variables	ASM uptake		Crude Odds Ratio (95% CI)	P-value	AOR (95% CI)	P-value
	Yes (%)	No (%)				
**Age (in years)**						
18–24	8 (53.3)	7 (46.7)	1		1	
25–34	60 (94.6)	38 (5.4)	1.38 (0.46, 4.12)	0.562	1.10 (0.32, 3.74)	0.883
35–44	104 (61.2)	66 (38.8)	1.38 (0.48, 3.98)	0.553	0.84 (0.25, 2.88)	0.786
≥45	93 (68.4)	43 (31.6)	1.89 (0.64, 5.56)	**0.246**	0.96 (0.28, 3.37)	0.954
**Residence**						
Urban	191 (59.9)	128 (40.1)	1.91 (1.16, 3.14)	**0.011**	1.37 (0.72, 2.62)	0.338
Rural	74 (74.0)	26 (26.0)	1		1	
**Marital status**						
Single	18 (58.1)	13 (41.9)	1		1	
Married	156 (63.4)	90 (36.6)	1.25 (0.59, 2.67)	0.562	0.91 (0.35, 2.35)	0.847
Divorced/Separated	42 (60.0)	30 (40.0)	1.01 (0.43, 2.37)	0.980	1.04 (0.36, 2.99)	0.937
Widowed	49 (70.0)	21 (30.0)	1.69 (0.70, 4.05)	**0.244**	1.64 (0.55, 4.85)	0.374
**Educational status**						
No formal education	65 (63.7)	37 (36.3)	1		1	
Primary education	101 (63.9)	57 (36.1)	1.01 (0.60, 1.69)	0.974	1.00 (0.55, 1.84)	0.999
Secondary education	58 (55.8)	46 (44.2)	0.72 (0.41, 1.26)	**0.245**	0.61 (0.29, 1.29)	0.192
College and above	41 (74.5)	14 (25.5)	1.67 (0.80, 3.45)	**0.169**	0.98 (0.33, 2.91)	0.970
**Occupational status**						
Government employee	62 (68.9)	28 (31.1)	1.77 (0.93, 3.37)	**0.082**	1.01 (0.44, 2.31)	0.974
Housewife	55 (56.7)	42 (43.3)	1.05 (0.57, 1.94)	0.882	0.75 (0.37, 1.51)	0.415
Merchant	59 (67.0)	29 (33.0)	1.63 (0.86, 3.10)	**0.138**	1.08 (0.50, 2.36)	0.841
Daily laborer	40 (55.6)	32 (44.4)	1		1	
Student/Farmer	31 (77.5)	9 (22.5)	2.76 (1.15, 6.61)	**0.023**	2.31 (0.77, 6.93)	0.137
Other	18 (56.3)	14 (43.7)	1.03 (0.44, 2.38)	0.948	0.57 (0.21, 1.52)	0.261
**Monthly income (Ethiopian birr)**						
≤2500	176 (61.1)	112 (38.9)	1		1	
2501–5000	60 (65.2)	32 (34.8)	1.19 (0.73, 1.95)	0.480	1.21 (0.71, 2.06)	0.487
≥5001	29 (74.4)	10 (25.6)	1.85 (0.87, 3.93)	**0.113**	1.79 (0.80, 4.02)	0.157
**ART facility catchment**						
Within the catchment	195 (59.8)	131 (40.2)	0.49 (0.29, 0.82)	**0.007**	1.73 (0.58, 5.18)	0.324
Out of the catchment	70 (75.3)	23 (24.7)	1		1	
**Distance to ART facility (in minutes)**						
≤60	187 (58.4)	133 (41.6)	1		1	
>60	78 (78.8)	21 (22.2)	2.64 (1.55, 4.49)	**<0.001**	2.90 (1.67, 5.04)	**<0.001**
**Duration on ART (in years)**						
≤5	46(51.1)	44(48.9)	1		1	
>5	219(66.6)	110(33.4)	1.90(1.19, 3.05)	**0.008**	2.21 (1.34, 3.64)	**0.002**
**Baseline WHO clinical stage**						
I	132(62.3)	80(37.7)	1.86(0.69, 5.01)	**0.222**	2.07 (0.70, 6.09)	0.186
II	51(62.2)	31(37.8)	1.85(0.65, 5.30)	0.251	1.71 (0.55, 5.27)	0.353
III	74(68.5)	34(31.5)	2.45(0.87, 6.90)	0.090	2.33 (0.77, 7.07)	0.134
IV	8(47.1)	9(52.9)	1		1	
**Recent viral load**						
Undetectable	254(64.0)	143(36.0)	1.78(0.75, 4.20)	**0.191**	1.63 (0.65, 4.11)	0.301
Detectable	11(50.0)	11(50.0)	1		1	
**Regimen Change**						
Yes	239(64.6)	131(35.4)	1.61(0.89, 2.94)	**0.118**	0.80 (0.33, 1.94)	0.616
No	26(53.1)	23(46.9)	1		1	
**INH prophylaxis**						
Completed	253(64.1)	142(35.9)	1		1	
Not complete/Not given	12(50.0)	12(50.0)	1.78(0.78, 4.07)	**0.171**	0.81 (0.34, 1.96)	0.645
**CPT prophylaxis**						
Ongoing taking	16(51.6)	15(48.4)	1		1	
Not complete	11(73.3)	4(26.7)	2.58(0.67, 9.88)	**0.167**	1.93 (0.45, 8.17)	0.374
Completed	179(68.6)	82(31.4)	2.05(0.97, 4.34)	**0.062**	1.42 (0.62, 3.26)	0.413
Not given at all	59(52.7)	53(47.3)	1.04(0.47, 2.31)	0.916	0.93 (0.39, 2.25)	0.879
**Missed clinical appointment**						
Yes	43(54.4)	36(45.6)	1		1	
No	222(65.3)	118(34.7)	1.58(0.96, 2.59)	**0.073**	1.91 (1.13, 3.25)	**0.016**
**Disclosure status**						
Yes	246(64.9)	133(35.1)	2.04(1.06, 3.94)	**0.032**	1.79 (0.89, 3.63)	0.104
No	19(47.5)	21(52.5)	1		1	
**Social support**						
Poor	118(54.9)	97(45.1)	1		1	
Intermediate	116(71.2)	47(28.8)	2.03(1.32, 3.13)	**0.001**	2.02 (1.29, 3.17)	**0.002**
Strong	31(75.6)	10(24.4)	2.55(1.19, 5.46)	**0.016**	2.71 (1.23, 5.97)	**0.013**

## Discussion

The present study aims to determine the uptake and associated factors of the six month MMS/ASM of care among stable adult clients on antiretroviral treatment. The overall prevalence of uptake of six month MMS/ASM was 63.25% (95% CI: 58.61%, 67.88%) and was significantly associated with no missed clinical appointment, distance to ART facility greater than sixty minutes, duration on ART greater than five years, and intermediate and strong social support.

The uptake of six month MMS/ASM of care (63.25%) found in this study is consistent with the study conducted in Conakry Guinea [15]. Our finding is higher than studies conducted in South Africa, Zambia, and Ethiopia [14, 16, 19], but lower than a previous study from Malawi and Tanzania [17, 18]. This might be due to the differences in the type of health facility, criteria for the eligibility of ASM, and health service delivery.

The current study revealed no missed appointments were positively associated with the uptake of six-month MMS/ASM of care. Clients who have not missed clinical appointments were more likely to uptake six month MMS/ASM of care as compared to those who have ever missed clinical appointments. The finding is supported by the studies conducted in Uganda and Zambia [16, 30]. The plausible explanation for the finding might be that the ASM of care has the potential to reduce unnecessary ART clinic attendance and this may result in support for adherence to treatment for the long term and improve health outcomes [31]. To maintain the visits for clinical appointments, health care facility management and the organizational system of health negatively affect the ability of ART clients [32]. Pieces of evidence from studies suggest that missed clinic appointments are associated with interruptions in ART, overlooked co-infections, lower CD4 counts, a raised risk for drug resistance, and virologic failure [33]. Thus, to reduce treatment interruptions and improve retention in care, it’s important for stable PLHIV to uptake the six month MMS/ASM of differentiated service delivery.

According to our findings, the uptake of six month MMS/ASM of care is higher among stable adult HIV clients on ART whose travel distance to the ART facility is >60 minutes compared to travel distance to the ART facility of ≤60 minutes. This might be due to the six months of clinical visits and medication refills having to reduce the frequency of longer travel time to ART facilities, and vice versa. It also reduces the transport cost and long waiting time in the facility for clients [6]. Thus, stable HIV-infected clients attending ART clinics from a longer distance should be encouraged to uptake the six month MMS/ASM of differentiated service delivery.

Duration of ART is another significantly associated factor in the uptake of six month MMS/ASM of care. Clients with a duration of ART >5 years are more likely to uptake six month MMS/ASM of care than those with a duration of ART ≤5 years, which is consistent with the previous studies conducted in Jimma and Northwest Ethiopia [19, 34]. The possible explanation could be that clients who stay longer duration on ART may understand and benefit from the ART service-related delivery of care approach.

Moreover, patients with intermediate and strong social support are more likely to uptake the six month MMS/ASM of care compared to those with poor social support. The finding is in agreement with the previous studies conducted in South Africa and Ethiopia [19, 27]. Strong social support among PLHIV was significantly associated with a higher level of health-related quality of life [35]. Therefore, strong social support may increase the utilization of HIV service delivery in PLHIV and ART.

### Limitations of the study

Due to the current study was based on data obtained through interviews and observation of clients’ medical records, some variables were incomplete in the record like baseline viral load and recent CD4 count. The nature of the study design we used cannot establish the causal effect relationship between the outcome and independent variable. As the study was institution-based, it doesn’t include different settings outside and is not generalizable for them.

## Conclusion

According to this study, the uptake of six month MMS/ASM of care was six out of ten stable clients on ART. Missed appointments, distance to ART facility, duration on ART, and intermediate and strong social support were factors significantly associated with the uptake of six month MMS/ASM. To further improve the uptake of six month MMS/ASM of care public health hospitals with supporting governmental and non-governmental organizations should design precise interventional measures that need to be undertaken on factors associated with the uptake of six month MMS/ASM of care to improve the uptake for ART provision expansion and HIV treatment targets achievement. Health professionals who provide ART services should strengthen the provision of counseling on the benefits of six month MMS/ASM of HIV care to stable clients on ART, especially those who missed clinical appointments and had poor social support. Further, a prospective cohort study should be conducted to assess the impact of six month MMS/ASM on the retention of clients on ART service.

## Supporting information

S1 Dataset The dataset supporting the findings of this study. (XLS)
